# Comparative physiological analyses and the genetic basis reveal heat stress responses mechanism among different *Betula luminifera* populations

**DOI:** 10.3389/fpls.2022.997818

**Published:** 2022-09-23

**Authors:** Xian-Ge Hu, Yilei Xu, Ning Shen, Mingtong Liu, Hebi Zhuang, Priyanka Borah, Zaikang Tong, Erpei Lin, Huahong Huang

**Affiliations:** The State Key Laboratory of Subtropical Silviculture, Institute of Biotechnology, College of Forestry and Biotechnology, Zhejiang A&F University, Hangzhou, China

**Keywords:** *Betula luminifera*, populations, physiological, heat stress, transcription factor

## Abstract

*Betula luminifera* is a subtropical fast-growing timber species with high economic value. However, along with global warming, heat stress become one of the main environmental variables that limit the productivity of *B. luminifera*, and the response of diverse geographic populations to high temperatures is still unclear. In order to offer a comprehensive understanding of the behavior of *B. luminifera* under heat stress, the physiological responses of six *B. luminifera* populations (across the core distribution area) were described in this work in an integrated viewpoint. The results showed that a multi-level physiological regulatory network may exist in *B. luminifera*, the first response was the activity of resistant enzymes [e.g., peroxidase (POD)] at a preliminary stage of 2 h heat stress, and then the proline (osmoregulation substance) content began to increase after 24 h of continuous high-temperature treatment. In addition, photosynthesis was stronlgly affected by heat stress, and the net photosynthetic rate (*P_n_*) showed a downward trend under heat treatment in all six *B. luminifera* populations. Interestingly, although the physiological change patterns of the six *B. luminifera* populations were relatively consistent for the same parameter, there were obvious differences among different populations. Comprehensive analysis revealed that the physiological response of Rongshui (RS) was the most stable, and this was the representative *B. luminifera* population. Illumina RNA-seq analysis was applied to reveal the specific biological process of *B. luminifera* under heat stress using the RS population, and a total of 116,484 unigenes were obtained. The differentially expressed genes (DEGs) between different time periods under heat stress were enriched in 34 KEGG pathways, and the limonene and pinene degradation pathway was commonly enriched in all pairwise comparisons. Moreover, transcription factors including bHLH (basic helix–loop–helix), MYB, WRKY, and NAC (NAM, ATAF1/2, and CUC2) were identified. In this study, the physiological response and tolerance mechanisms of *B. luminifera* under high temperature stress were revealed, which can conducive to the basis of *B. luminifera* selection and resistance assessment for cultivation and breeding.

## Introduction

*Betula luminifera* (H.) Winkl, belonging to the Betulaceae family, is a representative fast-growing timber plantation tree species (Chen, 2006; Zhang et al., 2010). *B. luminifera* is widely distributed in the subtropical regions and covers 14 provinces of China; the core distribution areas are mainly concentrated in Guizhou, Yunnan, Hunan, Guangxi, and Jiangxi provinces (Pan et al., 2017). This species is monoecious, mainly seed-propagated, and flowering is initiated after an average of 2 years (Cao and Fang, 2006). *B. luminifera* has an extremely high economic value among subtropical tree species because of its high-quality timber, short juvenile phase and fast growth (Li et al., 2018). In the subtropics, *B. luminifera* has become a recognizable landscape plant due to its high ornamental value in both garden and street landscaping. It’s essential for the development of *B. luminifera* plantations to multiply elite germplasm on a large scale, with high yield and resilience to biotic and abiotic challenges. However, there are disappointingly few publications on the physiological responses and resistance evaluation of *B. luminifera* under biotic and abiotic challenges, particularly the variations in response to stress among different populations.

As an important economic plant in subtropical regions, the wood quality of *B. luminifera* will be influenced through various environmental variables. Statistical evidence already implied that as a result of climate change, extremely high temperatures will occur more frequently (Skendžić et al., 2021; Iyakaremye et al., 2022), so heat stress become a growing threat to plant. Heat stress can trigger alters on morphological and physiological in plants, significantly influencing plant production and development (Allakhverdiev et al., 2008). Plants are sessile organisms, making them more susceptible to temperature changes. To counteract abiotic stress, plants may be forced to change their cellular state. In fact, plants have a series of capabilities to adapt to irregular increases in temperature, including basal thermotolerance, to make sure plants survive when exposed directly to extremely high temperatures and acquire thermotolerance, in which plants enhance their resistance to fatal heat stress following a period of preexposure to a nonlethal high temperatures (Li et al., 2021).

Nevertheless, a number of harmful occurrences, such as excessive reactive oxygen species (ROS) generation and the deterioration of cellular structural elements (Li et al., 2018), still occur when plants encounter extremely high-temperature environments. After exposure to high temperatures, plant photosynthesis is typically hindered, and heat stress makes the photosynthetic machinery vulnerable to damage (Wang et al., 2018). Additionally, it has been found that heat stress can alter the integrity of thylakoid membranes and the ultrastructure of chloroplasts (Allakhverdiev et al., 2008; Yamamoto et al., 2008), which greatly reduced plant thermotolerance. Based on the determination of physiological parameters under stress treatment, the response mechanisms of transcriptomics and metabolomics were analyzed for *Populus tomentosa* under heat stress (Ren et al., 2019); 20 coconut populations’ physiology response and resistance evaluation were obtained under low-temperature stress (Sun et al., 2021); and the physiological and transcriptomic response mechanism of *Santalum album* were revealed (Zhang et al., 2017).Therefore, the determination of physiological indexes is an effective means to assess plant responses to heat stress.

Populations with different adaptability are the foundation for breeding new varieties and creating new germplasm, and population selection and evaluation would advance the comprehension utilized of trees (Zhang et al., 2021). In this study, the physiological responses of six *B. luminifera* germplasm resources to heat stress were analyzed and assessed. The results showed that a multi-level physiological regulatory network may exist in *B. luminifera*, and the first response was the activity of resistant enzymes, and then the osmoregulation substance content began to increase after continuous high-temperature treatment. Furthermore, *B. luminifera* sequence transcriptomes under heat stress were obtained using second generation sequencing, and the differentially expressed genes (DEGs) and transcription factors (TFs) were identified and annotated. The analysis of heat resistance of *B. luminifera* populations will provide the basis for *B. luminifera* breeding, allowing for a greater diversity of resistant *B. luminifera* germplasms to fulfill current and future needs.

## Materials and methods

### Plant materials, growth conditions and treatments

**Figure 1:**
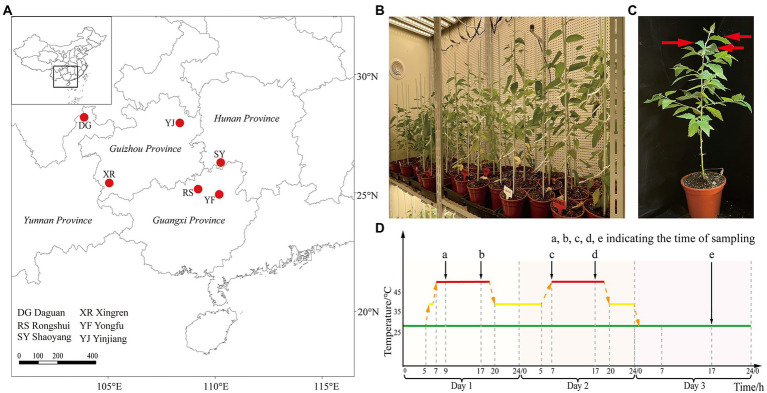
Plant material and experimental design. **(A)** The provenance locations of *B. luminifera* populations. **(B)** The growth chambers for the light/dark regime in heat stress treatment. **(C)** Sampling locations. Red arrows indicate sampling location. **(D)** Schematic diagram of the heat stress experimental design. The red line indicates the timeline of heat treatment, the green solid line indicates room temperature, and the black arrows (a, b, c, d, and e) indicate the time of sampling.

This study was performed at Zhejiang Agriculture and Forestry University (30.23°N, 119.72°E) in China. The germplasm resources (seeds) of *B. luminifera* provenances were collected from six typical suitable habitats in different provinces: Guangxi [Rongshui (RS) population (109.26°E, 25.08°N), Yongfu (YF) population (110.01°E, 25.01°N)], Hunan [Shaoyang (SY) population (111.28°E, 26.99°N)], Guizhou (Yinjiang (YJ) population (108.41°E, 27.99°N), Xingren (XR) population (105.19°E, 25.44°N)] and Yunnan (Daguan (DG) population (103.89°E, 27.75°N)], and were grown in the State Key Laboratory of Subtropical Silviculture in Zhejiang Province, China (Figure 1A). The seeds were germinated in transparent petri dishes with two layers of filter paper and then transplanted to seedling clods (Jiffy-7® –Peat Pellets and Coco Pellets) with a diameter of 30 mm. After growing for 2 weeks, they were transplanted to the soil with regularly watering in normal environment for 3 months. For adaptation, healthy seedlings (height, ~25 cm) were cultured in growth chambers for 7 days (at 25°C on a 12 h day/night cycle; light intensity, 250 μmol m^−2^ s^−1^) (Figures 1B,C). The relative value of humidity was kept at 65%.

Heat stress therapy consisted of raising the temperatures in growth chambers during the light/dark regime. We selected 12 individuals from each population for heat treatment. The treatment timeline was as follows: 25°C (room temperature); 25°C to 35°C, 0.5 h (warming); 35°C to 45°C, 0.5 h; and at 45°C during the day (Figure 1D). The sampling time for physiological index determination and RNA-seq analysis under heat treatment was 2, 10, 24, and 34 h, and recovery after 10 h (Figure 1D). The temperature setting in this study was inspired primarily by the previous heat treatment of *B. luminifera* (Pan et al., 2017) and seedling stage of other woody plants in response to heat stress (Chen et al., 2014; Ren et al., 2019). The third to fifth leaves from top to bottom were collected to measure photosynthesis-related parameters and physiological indexes (Figure 1C). Liquid nitrogen was used to freeze samples and stored at −80°C. To ensure repeatability of the results, the experiment was set up with three replicates, each consisting of four plants.

### Estimation of the proline (pro) content

Proline (Pro) is a typical osmotic adjustment substance, which is commonly used to estimate plant resistance to diverse abiotic stimuli (Zhang et al., 2017; Ren et al., 2019; Sun et al., 2021). Pro helps to adjust and maintain the cell osmotic balance, participates in decreasing cell redox potential reactions and is an important indicator of the heat response in plants. In this study, the Pro contents were measured with the commercially available assay kits (Nanjing Jiancheng Bioengineering Institute, Nanjing, CHN). To determine the Pro content, 0.1 g of leaf tissues were pounded to a powder combined with a homogenization medium, and centrifuged at 3,500 rpm and 4°C for 10 min. In accordance with the manufacturer’s instructions, the supernatant was then used for analysis. Pro content was calculated as g/g FW based on the mixture of 520 nm absorbance.

### Estimation of the antioxidant enzymes activities

The microplate reader was used to determine the activities of enzymes [peroxidase (POD), catalase (CAT), and superoxide dismutase (SOD)] by using commercially available assay kits (Nanjing Jiancheng Bioengineering Institute, Nanjing, CHN). To determine the activities of antioxidant enzymes, 0.15 g of fresh leaves frozen in liquid nitrogen were homogenized in 1.35 ml of physiological saline solution. After centrifuging the homogenized samples for 10 min at 4°C and 3,500 rpm, the supernatants were used to analyze enzymes activity. The specific steps were implemented as per the manufacturer’s instruction.

Utilizing xanthine and xanthine oxidase frameworks, the SOD activity was determined at 450 nm (Marklund and Marklund, 1974). One unit of SOD action was defined as the amount of enzyme needed to provide a 50% inhibition of the xanthine and xanthine oxidase system reaction during the extraction of 1 mg of protein per ml. The unit per milligram of protein used to describe SOD activity. The CAT activity was directed by detecting the decrease in absorbance at 405 nm caused by H_2_O_2_ breakdown (Luck, 1971). The assay principle was based on catalase’s reaction to breakdown H_2_O_2_, which absorbs maximum at 405 nm. Spectrophotometrically, the POD activity was determined at 420 nm by activating oxidation in the presence of H_2_O_2_ (Reddy et al., 1985). Purpurogallin, which has a wavelength of 420 nm, is produced in the assay by the reaction of peroxidase and pyrogallol. The quantity of enzyme required to catalyze the reaction of 1 g of substrate by 1 mg of tissue protein once every minute at 37°C was referred to as one unit of POD activity.

### Determination of photosynthetic parameters

Indexes of photosynthesis including the net photosynthetic rate (*P_n_*), stomatal conductance (*G_s_*), transpiration rate (*T_r_*), and intercellular CO_2_ concentration (*C_i_*) of the third and fourth fully expanded leaves of 3-month-old *B. luminifera* seedlings exposed to heat stress were measured. The photosynthetic parameters of the *B. luminifera* populations were determined by using LI-6800 photosynthetic system with standardized parameters. The instrument settings were: 800 μ mol m^−2^ s^−1^ photosynthetic photon flux density (PPFD), 55% relative air humidity, and 400 μmol mol^−1^ ambient CO_2_ concentration.

### RNA isolation, library construction, and sequencing

Based on comprehensive analysis of above physiological indexes, one population was defined as a representative for *B. luminifera* heat resistance evaluation and was used for second generation sequencing analysis to reveal the genetic basis of *B. luminifera* under heat stress. TRIzol Reagent (pre-chilled) (Invitrogen, Carlsbad, CA, United States) was used to extract total RNA from heat-stressed *B. luminifera* (three biological replicates each). The RNA concentration, quality and integrity were determined using a NanDrop 2000 (Thermo Fisher Scientific Inc., Waltham, MA, United States) and Agilent 2,100 Bioanalyzer (Agilent Technologies, Inc., Santa Clara, CA, United States), respectively (Liu and Zhu, 2020). A total of 15 libraries were separately produced from heat-treated samples at 2, 10, 24, and 34 h, and recovery after 10 h (three biological replicates for each sampling time point) using the TruSeq RNA Sample Preparation Kit (Illumina, San Diego, CA, United States) following the manufacturer’s instructions. Using poly-T oligo-attached magnetic beads, mRNA was extracted from total RNA. cDNA synthesis was implemented following our previous research (Hu et al., 2022). RNA sequencing was implemented by using Illumina NovaSeq 6,000 platform and 150 bp paired-end raw reads were generated.

### RNA-seq data processing and analysis

Raw reads in FASTQ format from second generation sequencing data of heat-stress samples were first treated to eliminate reads containing adapter sequences, reads with more than 10% unknown bases, and low-quality reads (i.e., where more than 50% of the bases in a read had a quality value Q ≤ 20). Using Trinity, a transcriptome *de novo* assembly was carried out (Grabherr et al., 2011) to obtain unigenes. Following assembly, BLASTx alignment between unigenes and the protein databases was carried out with a cut-off E-value of 10^−5^, in the following priority order: NCBI non-redundant protein sequences (NR); Gene Ontology (GO); Kyoto Encyclopedia of Genes and Genome (KEGG); evolutionary genealogy of genes: Non-supervised Orthologous Groups (eggNOG); Protein family (Pfam); Swiss-Prot (a manually annotated and reviewed protein sequence database). The best alignment results were utilized to establish the unigenes’ sequence direction.

Clean reads were mapped to the reference unigenes with Bowtie (Langmead et al., 2009), and RNA-Seq by Expectation Maximization (RSEM) was used to measure the levels of gene expression and obtain the number of read counts for each unigenes in each sample (Li and Dewey, 2011). The gene expression level was calculated using the fragments per kilobase million (FPKM) method (Mortazavi et al., 2008), and a bar graph was used to examine the number of transcripts among all heat stress samples after filtering. Based on the FPKM results, a correlation between samples was implemented by computing Pearson’s correlation for pairs of samples. Using the R package (DESeq 1.10.1) (Love et al., 2014), differential expression analysis was carried out. Genes detected by DESeq that displayed an adjusted *p*-value ≤0.01 were designated as DEGs (Zhang et al., 2019). In addition, the heat-induced TFs were screened to identify highly expressed TF genes during heat stress.

### Quantitative RT-PCR analysis

RNA was extracted from heat-treatment *B. luminifera* after 2, 10, 24, and 34 h of heat stress and 10 h of recovery. RNAs were processed with DNase I to eliminate genomic DNA contamination using the TurboDNA-free Kit (Ambion, Austin, TX, United States). Then total RNA from each sample was reverse transcribed by PrimeScript™ RT Reagent Kit (Perfect Real Time; Takara) and used as a templet. A large ribosomal subunit 39 gene (RPL39) was selected as an internal reference gene. The gene-specific primers were presented in Supplementary Table S1. Melting curve analysis was implemented for each primer pair prior to the next analyses. qRT-PCR was implemented with the TB Green Premix Ex Taq II (Tli RNaseH Plus; Takara) on CFX96 real-time PCR detection system (CFX96; Bio-Rad, United States). The expression pattern was assessed by our previous protocol (Hu et al., 2022). For each gene and sample, the qRT-PCR data were acquired from three biological and three technical repeats.

## Results

### The difference of pro content among different populations under heat stress

**Figure 2:**
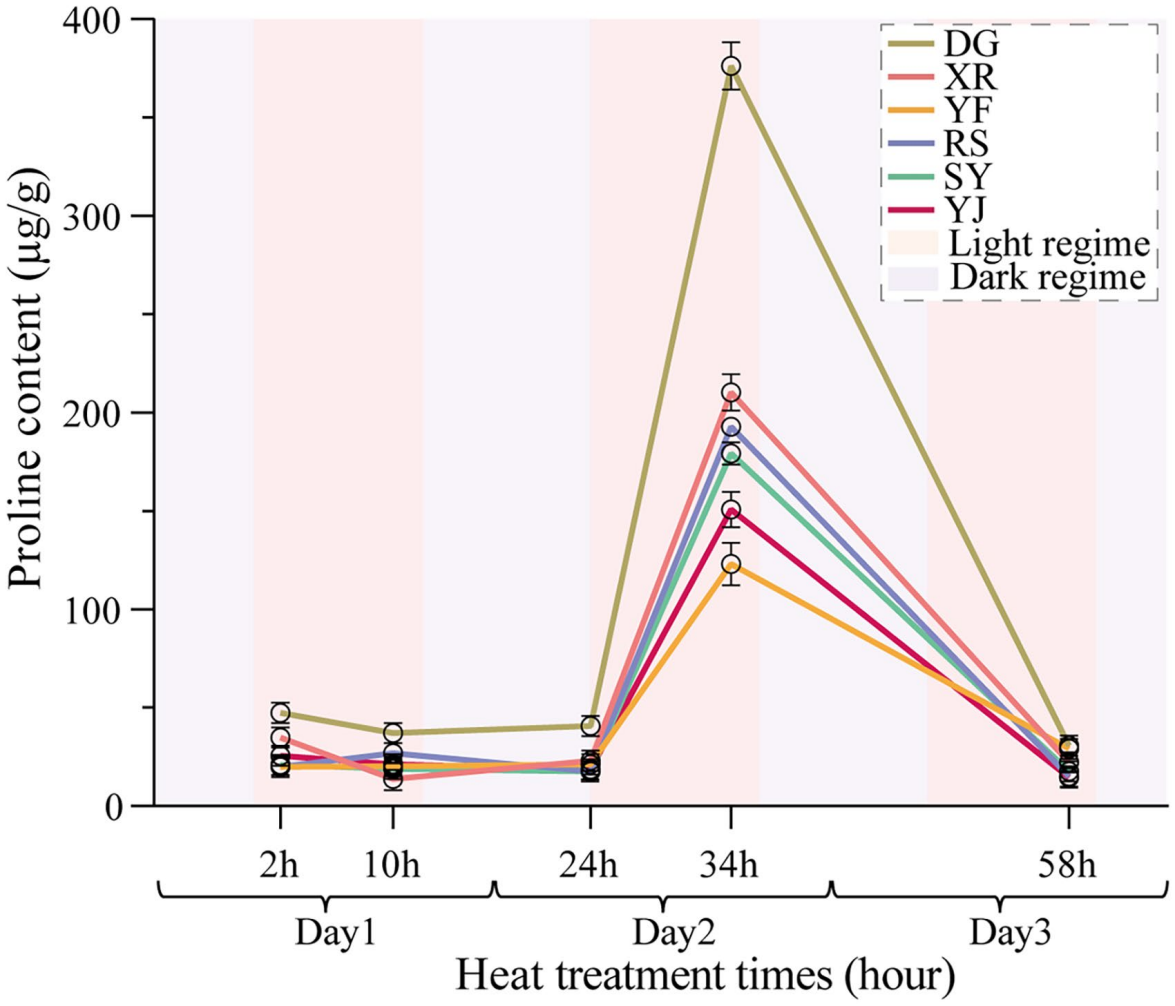
Effects of heat stress on *B. luminifera* populations’ pro contents under different temperature treatments times.

The Pro accumulation in the six *B. luminifera* populations fluctuated slightly during the preliminary stage of heat stress (2 h), and increased sharply after 24 h of heat treatment (Figure 2). Interestingly, the maximum value of the Pro content for the six populations all appeared at 34 h of heat treatment. However, the fold change had a significant difference among the *B. luminifera* populations, e.g., the Pro content in the DG population was more than 1.9-fold higher than that in the other populations at 34 h of heat treatment. According to the change pattern of the Pro content in each population under heat treatment, the DG population was the fastest to respond to the heat treatment, followed by the XR, RS, SY, YJ, and YF populations, of which YF was the slowest and had a relatively mild response to the heat treatment. As the temperature returned to normal, the Pro content gradually decreased and returned to normal levels (Figure 2). Therefore, the increased Pro level at high temperature was beneficial for enhancing plants’ endurance during the processing period, and the Pro content is an important indicator to evaluate *B. luminifera* heat resistance.

### The difference in defense enzyme activities among different populations under heat stress

**Figure 3:**
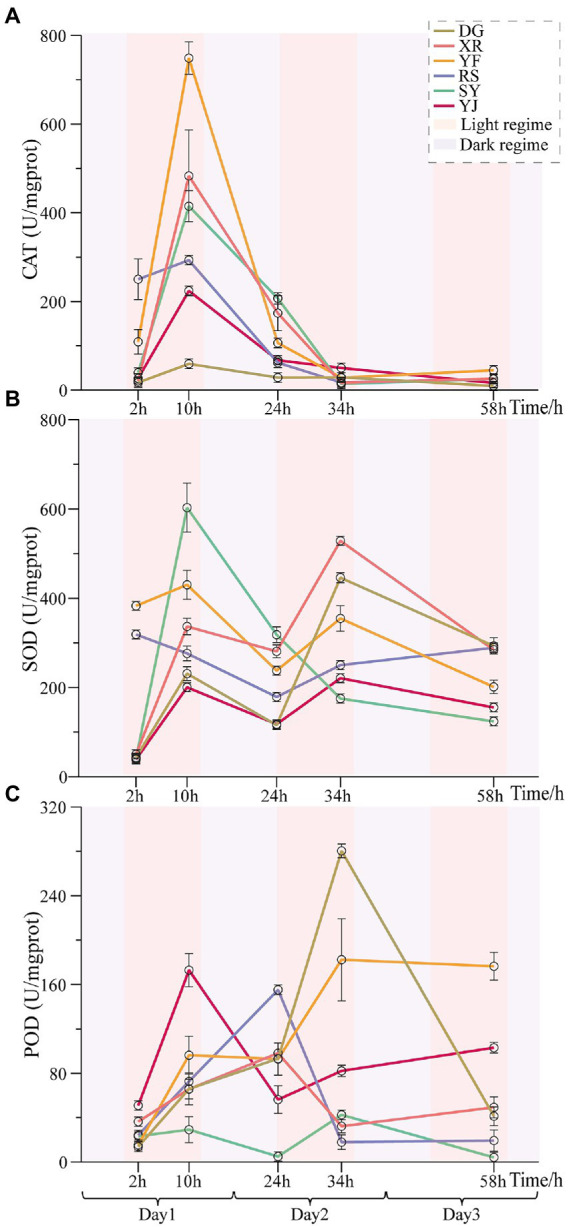
Effects of heat stress on POD, CAT, and SOD activities of *B. luminifera* populations under different temperature treatments times. **(A)** The change pattern of SOD activities. **(B)** The change pattern of POD activities. **(C)** The change pattern of CAT activities. Error bars indicating standard error (SE).

It was determined that the defense enzyme (SOD, CAT and POD) activity in the leaves of various populations of *B. luminifera* initially rose with temperature and time (Figure 3). In the first photoperiod under heat stress, the activities of almost all defense enzymes increased, and significant differences were found among the different *B. luminifera* populations. With the arrival of the dark cycle (temperature decreased to 35°C), the activities of these enzymes showed decreases (Figure 3). However, the enzyme activity continued to increase when the second stage of photoperiod began. In addition, irrespective of the light or dark regime, the activities of the defense enzymes (SOD, CAT, and POD) in the *B. luminifera* populations in response to heat treatment differed significantly. For example, although the SOD activity showed an increasing trend in the six populations in response to the heat treatment, the increase in the SY population was almost three-fold higher than that in the YJ population after 10 h of heat treatment. Overall, under heat treatment, except that the SOD activity of the SY population decreased continuously after reaching the maximum at 10 h of heat treatment, the change pattern of most of the *B. luminifera* populations increased with the extension of the heat treatment time but decreased appropriately during the dark cycle.

In our study, the change pattern of POD was almost consistent with that of SOD in the six *B. luminifera* populations, but the CAT activity of these populations decreased continuously after reaching the maximum at 10 h of heat treatment. In addition, even though the response of these defense enzyme activities in the *B. luminifera* populations to heat treatment were differed significantly, the change patterns of same enzyme activity among the six provenances were relatively consistent. The YJ and SY populations had the smallest and largest change ranges in SOD activity. The smallest and largest changes in POD activity occurred in the SY and DG populations respectively, and the YF and DG populations had the smallest and largest changes in CAT activity. In general, the changes in these three defense enzyme activities in the RS population were relatively stable.

### Effects of heat stress on the photosynthetic parameters of the *Betula luminifera* populations

**Figure 4:**
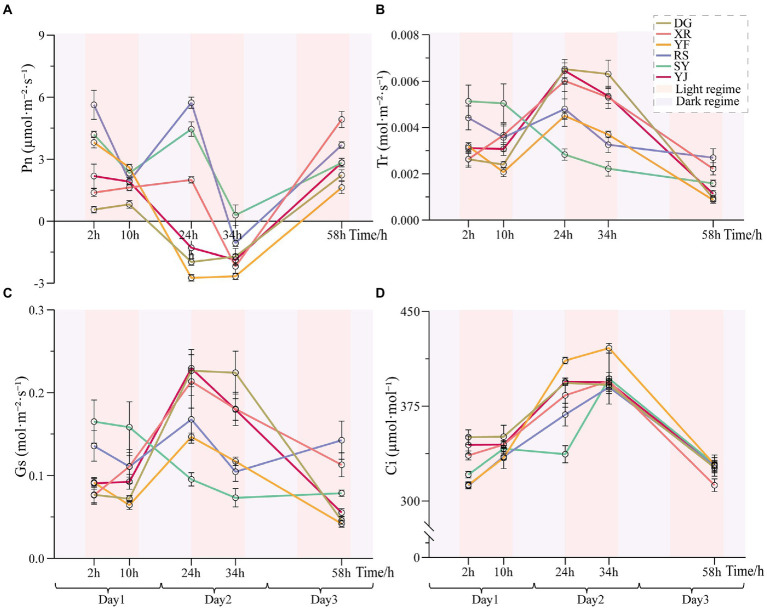
Photosynthetic parameters of *B. luminifera* populations under heat treatments. **(A)** Net photosynthetic rate (*P_n_*). **(B)** Transpiration rate (*T_r_*). **(C)** Stomatal conductance (*G_s_*). **(D)** Intercellular CO_2_ concentration (*C_i_*). Error bars indicating standard error (SE).

The net photosynthetic rate (*P*_n_) of leaves can directly reflect the strength of photosynthesis. The *P*_n_ values of the six *B. luminifera* populations decreased under heat stress (Figure 4). The main differences in *P*_n_ among the *B. luminifera* populations were that the RS, SY and YF populations decreased significantly after 10 h of heat treatment (decreased by 65, 55, and 30% compared with the preliminary stage of 2 h of heat stress); the YJ and DG populations began to decrease after 24 h of heat treatment (decreased by 160 and 170%); and the XR population did not decrease until 34 h of heat treatment (decreased by 167%). Although the *P*_n_ of the RS and SY populations decreased significantly at the preliminary stage of heat stress, *P*_n_ increased again after the dark cycle (35°C). Therefore, based on the change trend of the net photosynthetic rate under heat stress, the photosynthetic parameters of the YF population were the most sensitive to temperature treatment, and *P*_n_ changed regularly with temperature increases and time. However, the *P*_n_ of the XR population was relatively stable and recovered rapidly when heat stress was suspended, indicating its strong heat resistance.

Leaf stomatal conductance (*G*_s_) is the reciprocal of leaf stomatal resistance, which reflects the ability of plants to exchange CO_2_ and water vapor, and is one of the important physiological indicators affecting *P*_n_. Except for the XR population, the leaf *G*_s_ of the *B. luminifera* populations decreased at the preliminary stage of heat stress. In addition, except for the SY population, although the *B. luminifera* populations decreased during the heat stress, *G*_s_ was restored during the dark cycle (35°C). The transpiration rate (*T_r_*) of leaves is also one of the important factors affecting *P_n_*. In this study, the total dynamics of *T_r_* in the *B. luminifera* populations under heat stress had a similar pattern as *G_s_* (Figures 4B,C); one possible reason is that the opening or closing of stomata is the main factor affecting the transpiration rate. The intercellular CO_2_ concentration (*C_i_*) of the *B. luminifera* populations had a clear upward pattern with the increase of temperature and time, and decreased with the cessation of the heat stress. After 34 h of heat treatment, the greatest increases in the YF population were 33%, and the RS population had the smallest change, an increase of 26%.

### Transcriptomic changes of *Betula luminifera* in response to heat stress

Significant distinctions existed between the physiological responses of the various *B. luminifera* populations to heat stress, while RS was the most stable and representative population based on comprehensive analysis of the above physiological parameters. Therefore, this representative population was selected to reveal the genetic basis of *B. luminifera* under heat stress. A total of 705,350,026 reads (Supplementary Table S2) were generated from the heat-stressed RS population by using the Illumina sequencing platform. After quality checks and data cleaning, 653,379,564 clean reads (Supplementary Table S2) were obtained. Based on the remaining high-quality clean reads, Trinity produced 364,563 transcripts with an average length of 1,198 bp, and 116,484 unigenes with an average length of 1,021 bp were obtained from these transcripts. The N50 and N90 sizes of the transcripts and unigenes were 1844 and 497 bp, 1,598 and 420 bp, respectively (Table 1). The total length of all unigenes was 118,960,472 bp (Table 1; see Supplementary Figure S1 for the length distribution of all unigenes). Additionally, comparisons with our previous transcriptome assemblies of *B. luminifera* (Cai et al., 2018) demonstrated that the properties of the *B. luminifera de novo* transcriptome assembly were of high quality.

**Table 1:** Summary data of the assembled transcripts and unigenes in *B. luminifera*.

	Transcripts	Unigene
N50 (bp)	1844	1,598
N90 (bp)	497	420
GC (%)	39.54	38.24
Sequence number	364,563	116,484
Maximum length (bp)	17,354	17,354
Mean length (bp)	1197.78	1021.26
Total length (bp)	436,667,824	118,960,472

### Functional annotation of the *Betula luminifera* unigenes

**Figure 5:**
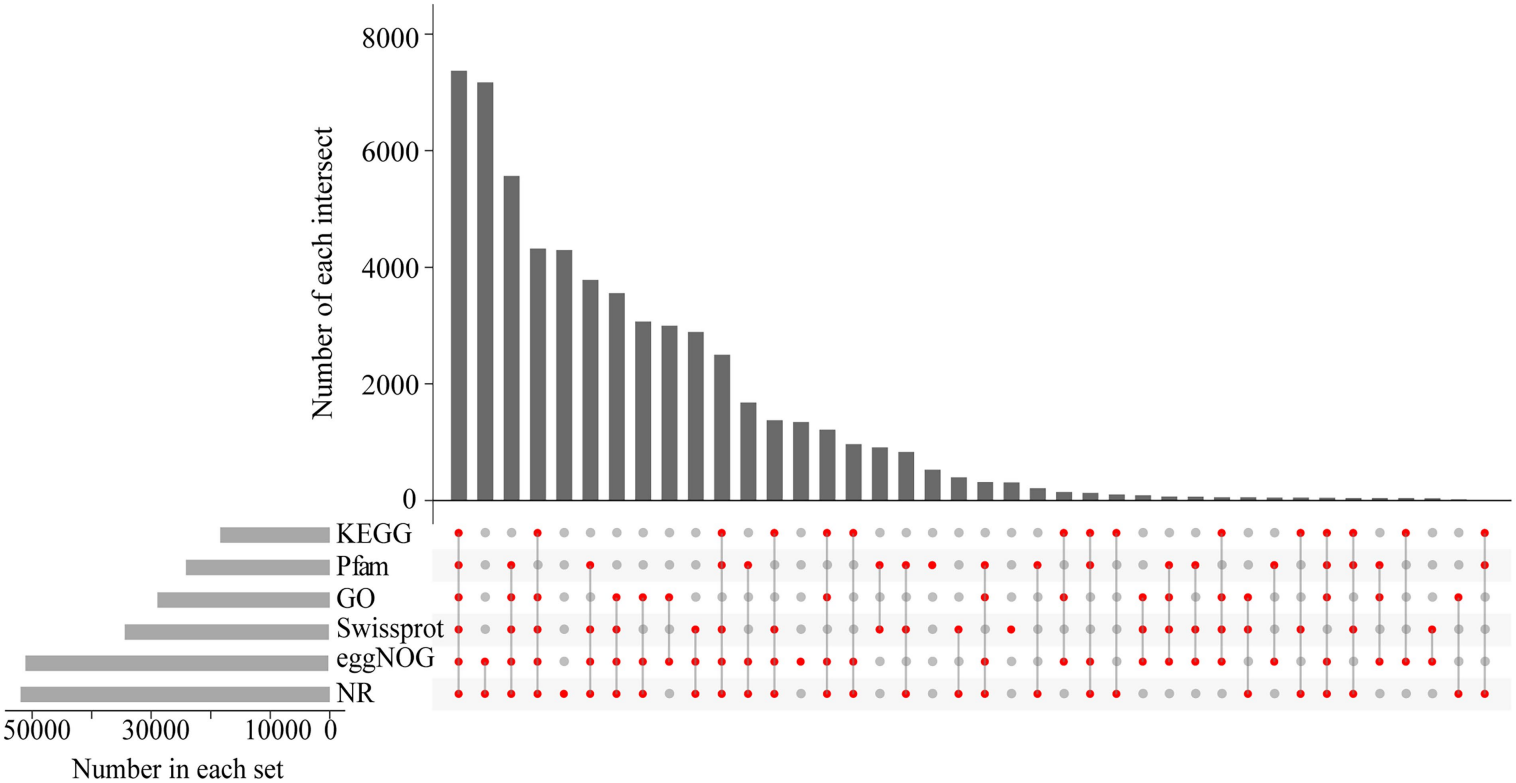
The unigenes homology searches against the protein databases.

**Figure 6:**
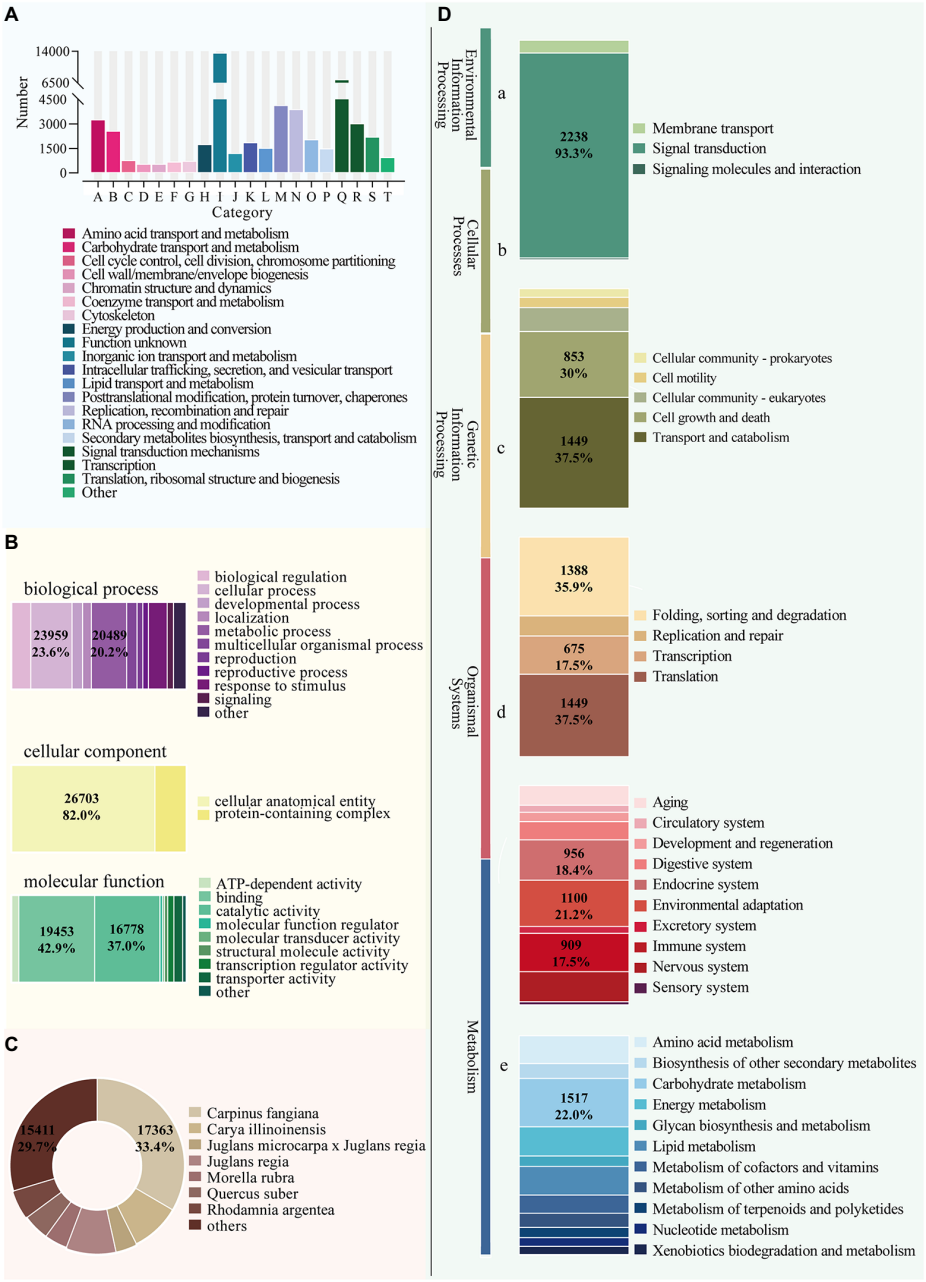
Functional annotation of *B. luminifera* unigenes generated by illumine sequencing. **(A)** Functional classification of unigenes by eggNOG analysis. **(B)** Functional classification of unigenes by GO analysis. **(C)** Functional annotation of unigenes by NR analysis. **(D)** A total of 21,184 unigenes were assigned to different KEGG terms. Different color blocks represent different terms, from top to down, “Environmental Information Processing” (a), “Cellular Processes” (b), “Genetic Information Processing” (c), “Organismal Systems” (d), and “Metabolism” (e).

We performed functional annotation using BLAST by comparing sequences against six databases (NR, GO, eggNOG, KEGG, Swiss-Prot, and Pfam) and received annotation results for 116,484 unigenes. In total, 58,650 unigenes were annotated in at least one database, with 7,370 annotated in all databases (Figure 5). The eggNOG database assigned a total of 50,872 unigenes to 26 functional clusters, with “signal transduction mechanisms” being the largest category (Figure 6A). According to three main categories (molecular function, cellular component, and biological process), GO annotation classification was carried out to further categorize the *B. luminifera* transcripts (Figure 6B). For biological process classification, “reproduction” (26,703 unigenes), “cellular process” (23,959) and “sulfur utilization” (20,489) were the three major categories. The major cellular component categories were “protein-containing complex” (208), “cytoskeletal motor activity” (198) and “cellular anatomical entity” (190). Unigenes involved in “cytoskeletal motor activity” (156), “catalytic activity” (125) and “structural molecule activity” (69) were highly represented in molecular function subgroups (Figure 6B).

Based on blasting to other species in NR database, we also discovered that the sequences of *B. luminifera* and allied species shared similar functional information. *Carpinus fangiana* had the highest quantity of hits to *B. luminifera* (17,367), followed by *Carya illinoinensis* (4782) and *Juglans regia* (4761) (Figure 6C), indicating a high degree of homology between birch and walnut. The unigenes were discriminated through the KEGG database to explore the function of these genes involved in biological pathways. A number of 18,387 unigenes were sorted into five major KEGG functional catgaries (Figure 6D). A substantial majority of isoforms, including “Carbohydrate metabolism” (1517), “Energy metabolism” (919), and “Lipid metabolism,”(903) were dispersed in “Metabolism” category (6881) (Figure 6D).

### Differential gene expression and functional enrichment analysis among different time periods

**Figure 7:**
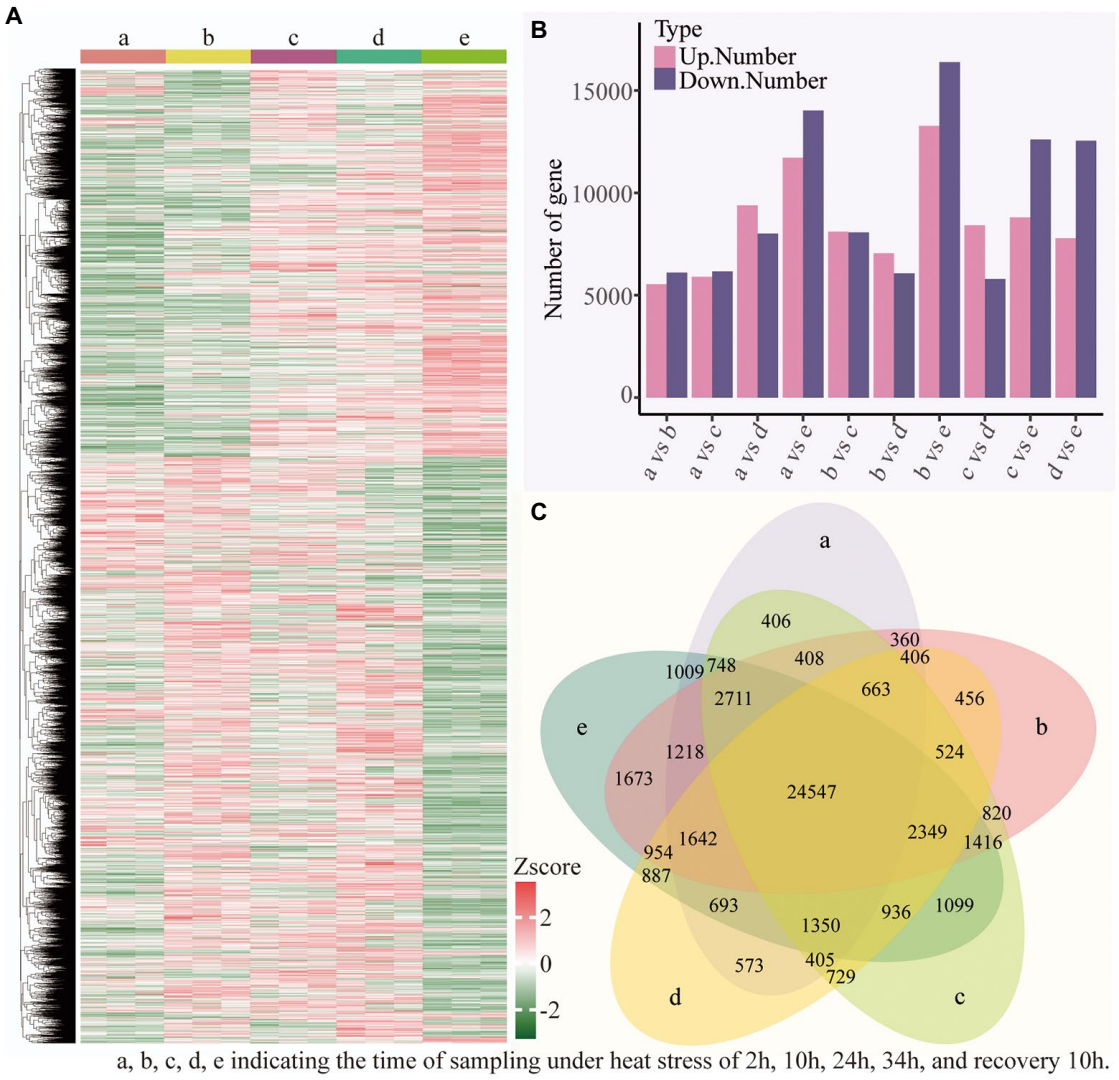
Transcriptional variations in *B. luminifera* under different treatments time period. **(A)** Hierarchical clustering analysis of the DEGs under different treatments time period. **(B)** The number of up- and down-regulated genes in different treatments time period. **(C)** Venn diagrams showed the proportions of the DEGs in different treatments time period.

To explore the specific biological process of heat resistance in *B. luminifera*, the transcriptional level of all genes modulated by the temperature stress was measured based on Illumina platform. The expression patten of these DEGs between all 10 sample pairs was shown in Supplementary Figure S2. In total, 38,925 DEGs were identified in the heat treatment (Supplementary Table S3); gene clustering was utilized to classify DEGs with similar expression patterns for the subsequent analyses, and the FPKM values of various genes were utilized for hierarchical clustering analysis (Figure 7A); and the detailed information of up- and down-regulated genes among different time periods is shown in Figure 7B. For example, between 2 h and 10 h of heat treatment, a total of 11,647 DEGs, i.e., 5,539 up- and 6,108 down-regulated DEGs, were identified. In addition, compared to the preliminary stage of heat stress (2 h), the number of DEGs (both up- and down-regulated genes) increased with the extension of the treatment time (heat stress of 10, 24, and 34 h, and recovery 10 h). Furthermore, we compared five datasets using Venn diagrams (Figure 7C), and there were 24,547 DEGs common to the 10 comparative sampling time periods.

**Figure 8:**
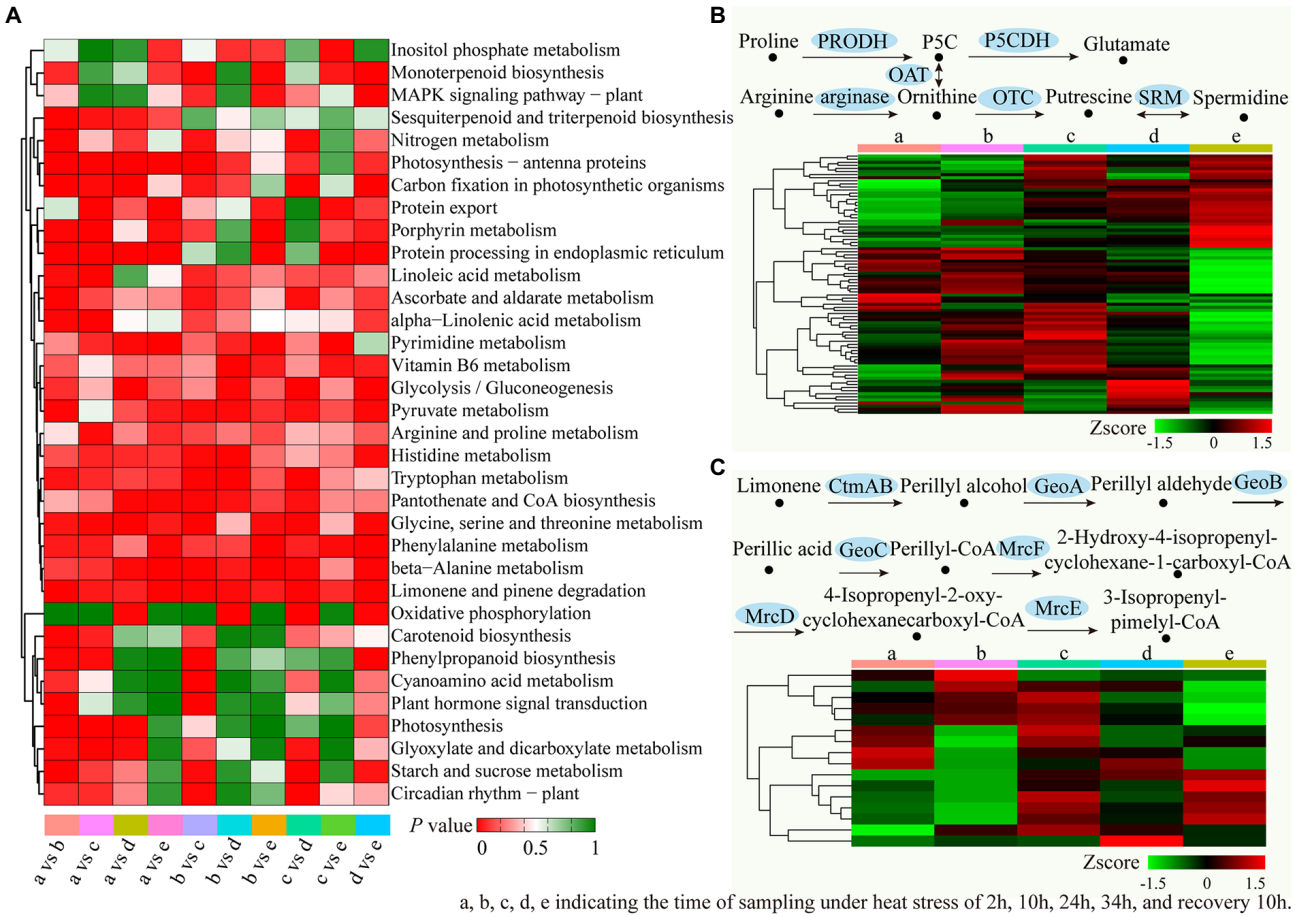
Enriched KEGG pathways analysis of the DEGs in different treatments time period. **(A)** The significant *p* values of each KEGG term in different treatments were shown by a heatmap. Red indicated significantly enriched KEGG terms. **(B)** Expression analysis of the unigenes related to the “arginine and proline metabolism” pathway. **(C)** Expression analysis of the unigenes related to the “Limonene and pinene degradation” pathway.

By analyzing the enriched KEGG terms, we had elucidated the biological functions of these DEGs. It was identified that 34 enriched KEGG pathways were identified for at least one duration of treatment (Figure 8A). When compared with the preliminary stage of 2 h heat stress, pathways (a), “Limonene and pinene degradation,” “Photosynthesis-antenna proteins” and “Protein processing in endoplasmic reticulum” showed the highest enrichment. Compared to 10 h of heat stress (b), 12 enriched KEGG pathways were identified, the top 3 pathways were “Limonene and pinene degradation,” “Pyruvate metabolism” and “Photosynthesis-antenna proteins.” Compared to 24 h of heat stress (c), “Limonene and pinene degradation,” “Phenylalanine metabolism” and “Glycine, serine and threonine metabolism” were the top 3 pathways. Compared to 34 h of heat stress (d), the top three enriched pathways were “Limonene and pinene degradation,” “beta-Alanine metabolism” and “Pyruvate metabolism.” Compared to the recovery samples (e), “Limonene and pinene degradation,” “Phenylalanine metabolism” and “Protein processing in endoplasmic reticulum” showed the highest enrichment. Furthermore, “Limonene and pinene degradation” was markedky enriched in all time periods during the heat treatments. Figures 8B,C displayed the DEG expression profiles for the “Arginine and Proline Metabolism” and “Limonene and Pinene Degradation” pathways, respectively.

**Figure 9:**
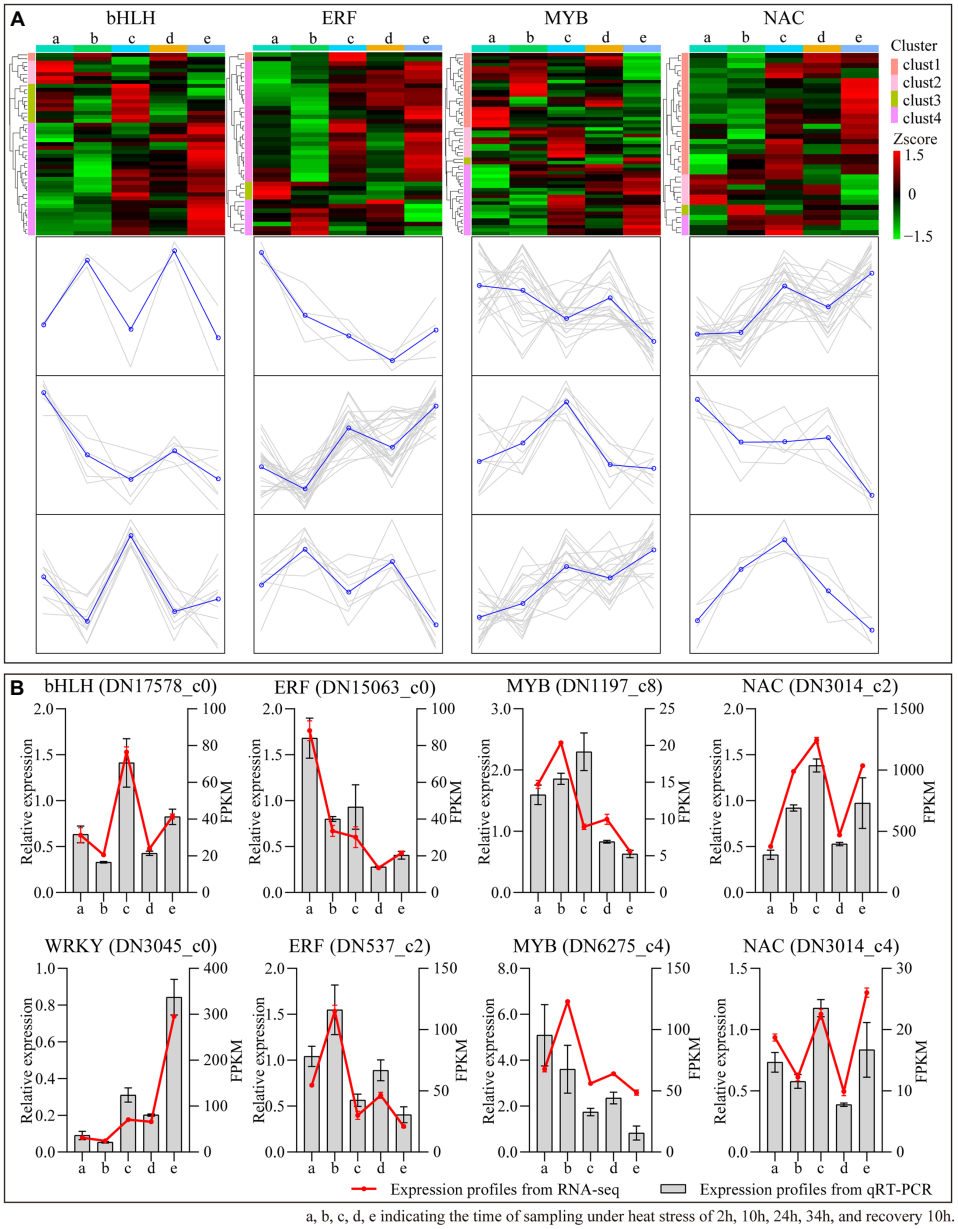
The differential expression pattern of the four TF families (MYB, NAC, and WRKY). **(A)** The differential expression pattern of the four TF families were depicted by a heatmap. **(B)** Validation of expression pattern of several TF genes by qRT-PCR.

TFs have important functions in many facets for plant growing, as well as in the face of heat treatment. In this study, iTAK software (Zheng et al., 2016) was employed to forecast the TFs of *B. luminifera* under heat stress. A column diagram depicts the numbers of the top 20 largest TFs families annotated between 10 comparative sampling durations (Supplementary Figure S3). The top three transcription factor families among the 10 comparative sampling groups belong to the bHLH, ERF, MYB and NAC families. The expression profiles of these four TF families at different heat treatment times are presented in a heatmap (Figure 9A). Most of the genes in these families belong to three types of expression patterns. To further evaluate the second-generation sequencing data, eight transcription factor genes were chosen at random to perform qRT-PCR (Figure 9B). The gene expression profiles were comparable with the FPKM values based on second generation sequencing, confirming the reliability of the sequencing data.

## Discussion

### Content of osmotic adjustment substances in response to heat stress

In this study, the change in Pro differed significantly among the six *B. luminifera* populations; the DG and XR populations had higher contents under heat treatment, which may indicate those populations are more sensitive to heat stress. Pro is generally implemented in estimating plant tolerance to a variety abiotic stimulus since it contributes to control and preserve cell osmotic equilibrium and serves a key role in lessening cell redox potential responses (Misra and Saxena, 2009; Yan et al., 2015). Pro performs as a remarkably powerful non-enzymatic antioxidant implicated at plant cells’ antioxidant defense (Gill and Tuteja, 2010). Additionally, heat-tolerant woody species like *P. tomentosa* and *Jatropha curcas* have also shown an increase in the Pro content of their tissues (Silva et al., 2010; Ren et al., 2019). In keeping with earlier reports, it was found that all six populations of *B. luminifera* had an increase in Pro content over the course of the heat stress’s 24 h, indicating that *B. luminifera* can withstand short-term heat stress.

Pro production is a general physiological response to high-temperature treatment, representing heat stress play a negative function on plant growth, and the increased osmotic substances under high stress was advantageous to enhancing the resistance of plants with the processing time (Lv et al., 2011). The likely mechanism of Pro accumulation in *B. luminifera* leaves in the stimulus of heat stress treatment was that the generation rate of ROS gone up, membrane lipid peroxidation raised, and cell membrane lipids degraded, leading to leaves amassing considerable levels of Pro (Wang et al., 2017). In addition, the important Pro synthesis genes were identified in the arginine and Pro metabolism pathways (Figure 8B). Therefore, *B. luminifera* responds to heat stress most likely by regulating proline synthesis genes (e.g., P5CS) to increase Pro levels (Supplementary Table S3). The raised Pro content at high temperatures aided in enhancing *B. luminifera* resistance. Based on the change pattern of the Pro content in the six *B. luminifera* populations under heat stress, Pro can be viewed as a key indicator for assessing *B. luminifera* tolerance, and this result agrees well with previous heat treatment studies on woody plants (Chen et al., 2014; Ren et al., 2019).

### Effects of heat stress on antioxidant activity

In this research, the increased antioxidant enzyme activities (SOD, POD, and CAT) suggested that cellular damage occurred when the *B. luminifera* populations were exposed to heat stress (45°C). Normally, oxidant–antioxidant balance exists in plants, which is critical for survival. However, heat stress causes ROS to be produced in greater abundance than normal (threshold value), which causes oxidative damage (Baxter et al., 2014). In response, plants can produce more antioxidant enzymes (e.g., POD and CAT) to deactivate or eliminate ROS (Ciou et al., 2011; Jin et al., 2011). Similar studies have been reported in other woody plants, e.g., *P. tomentosa* and *Jatropha curcas* (Silva et al., 2010; Ren et al., 2019). The same temperature treatment setting (45°C) were set to identify the heat-stress responsive miRNAs to disclose the miRNA-mediated regulatory network of *B. luminifera* under high temperatures (Pan et al., 2017).

Nevertheless, there were obvious differences in the responses of the different *B. luminifera* populations to heat treatment. Similar research results have also been reported for *Betula alnoides*, a related species of *B. luminifera*: the physiological indexes such as the leaf conductivity, net photosynthetic rate, transpiration and stomatal conductance were significantly different among populations under high-temperature treatment (45–50°C) (Chen et al., 2003). Overall, based on the change pattern of antioxidant enzyme (SOD, POD and CAT) activities under heat stress, the response of the RS population was in an intermediate fluctuating state among the six *B. luminifera* populations. Therefore, for the response of *B. luminifera* under heat treatment, the RS population was the representative of the six populations. In addition, physiological responses of different geographical populations to stress treatments that were significantly different have also been reported for other woody plants such as *Cocos nucifera* (Sun et al., 2021), *Azadirachta indica* (Zheng et al., 2008), and *Quercus variabilis* (Zhang et al., 2017). Therefore, analyzing the tolerance of *B. luminifera* seedlings from different populations under heat stress is of great help to select provenances with excellent resistance and to find artificial measures to improve the survival of seedlings.

### Effects of heat stress on photosynthesis

In this study, heat stress had a significant impact on photosynthesis in all six *B. luminifera* populations. Particularly, the *P_n_* was less than zero along with the extension of the heat treatment time. It is widely known that *P_n_* value is equal to total photosynthetic rate subtracting respiration rate, thus *P_n_* is the superimposed effect of photosynthesis and respiration. The *P_n_* become negative in this study, probably because the photosynthetic rate of *B. luminifera* seedlings was less than the respiration rate due to the damage of high temperature stress. This phenomenon also occurs in other plants under stress, the photosynthesis of plant leaves was reported to be inhibited beyond the optimal temperature range (Ashraf and Harris, 2013), other instances have included in *Anthurium* (Chen et al., 2021) and *Euphorbia pulcherrima* (Feng et al., 2009). Heat stress, in fact, will reduce plant photosynthetic capacity by causing damage to any component of the light-harvesting process, electron transport, and exploiting of assimilates in photosynthesis (Kurek et al., 2007; Sharkey and Zhang, 2010), especially for PS II, the photosynthetic system’s most heat-sensitive component (Song et al., 2014). Several crucial subunits (e.g., Psb27/28) and cofactors (e.g., CP43) involved in photosynthetic electron transport are required for PS II repair throughout heat stress (Liu et al., 2011; Sakata et al., 2013), and light-harvesting-related proteins (LHCs) are also of essential for light harvesting and photoprotection (e.g., LHC sub-classes of Lhcb1-8) (Alboresi et al., 2008).

Our results also showed that there were notable variations in heat-stress responses among the different *B. luminifera* populations. Under heat stress, in the initial stage of heat treatment, the *P_n_* values of the RS, SY, and YF *B. luminifera* populations decreased, whereas those of the DG, XR and YJ populations remained stable. In the second photoperiod, there was a significant decrease in *P_n_* in all six populations relative to the initial stage of heat treatment. In addition, the SY and RS populations’ photosynthesis recovered after a dark period, but the *P*_n_ also decreased in the new photoperiod. Interestingly, after the heat treatment stopped, *P_n_* basically recovered, indicating that although the photosynthesis of *B. luminifera* was sensitive to heat treatment, it still had a certain degree of heat resistance. In addition, many related genes involved in the photosynthetic electron transport system in *B. luminifera* were enriched (Figure 8), indicating that the photosynthetic electron transport and photophosphorylation of *B. luminifera* were affected by heat stress. Overall, our results suggest that the photosynthesis of *B. luminifera* may be damaged under high temperature stress, caused to inhibition of the photosynthetic process in *B. luminifera*.

### TFs involved in the response to high temperature treatment

In this study, we also performed transcriptome analysis on a representative population (RS) to reveal the genetic basis of *B. luminifera*’s self-regulation under heat stress. We established an approximately 98.0 GB-sized transcriptome data from *B. luminifera* heat-stressed leaves assembled into 116,484 unigenes, of which 44% were annotated against public databases. Our study will supply a substantial public data set and will assist functional gene researches of this important timber species. It is generally considered that when exposed in changing external temperatures, plants will increase their resistance through controlling gene induction and expression responded stress. According to the present study, 38,926 heat-inducible DEGs were identified. This dominance of gene expression under high temperatures shows a sustained adaptation during an extended time periods. In the public database, more than 93% of the DEGs from *B. luminifera* were annotated as homologs, suggesting that they are heat-stress responsive genes with previously reported homologs from other plants. Deeper study into these genes may reveal complete biological process underlying *B. luminifera* population responses to heat stress.

A series of TFs, e.g., bHLH, WRKY, NAC, and MYB, were identified and significantly differentially expressed with the extension of the high-temperature treatment time. In previous studies, TFs were shown to involved in stress-responsive gene transcriptional by recognizing DNA through sequence-specific pattern (Chen et al., 2014; Song et al., 2014; Ren et al., 2019). For instance, in *Manihot esculenta*, *WRKY*79 and heat shock TF 20 (*Hsf*20) enhanced melatonin biosynthesis through interacting with the promoter of N-acetylserotonin O-methyltransferase 2 (*ASMT*2) to increase disease resistance (Wei et al., 2017). In tomato, TF heat shock factor A1a (*HsfA1a*) promoted transcript accumulation of the melatonin biosynthesis gene *COMT1*, which strengthens plant resistance to cadmium (Cd) as a result (Cai et al., 2017). Therefore, the identification of TFs under stress is the key to understand the plant self-regulation network, and our research provides a genetic basis for revealing the adaptability of *B. luminifera* under heat stress.

## Conclusion

In conclusion, our results demonstrated a large set of parallel changes in physiological responses (photosynthetic parameters, osmolytes and antioxidant enzymes) in *B. luminifera* populations to heat stress. The greatly differences in adaptability among different populations, meaning that there is great potential to breeding new varieties with high heat-resistance for *B. luminifera*. On the other hand, among different *B. luminifera* populations, the consistent physiological response was the activity of resistant enzymes change firstly, and then the osmoregulation substance content began to increase after continuous heat stress. In addition, the current study created an abundant transcriptome for the leaves of *B. luminifera* that respond to heat stress. And comprehensive physiological and transcriptomic analysis integrated with qRT-PCR were performed to provide fundamental knowledge about biological changes in *B. luminifera* populations under heat treatment. In general, our research provided a valuable insight into the heat resistance of *B.luminifera* populations, which can conducive to the foundation of *B. luminifera* selection and resistance evaluation for cultivation and breeding.

## Data availability statement

The datasets presented in this study can be found in online repositories. The names of the repository/repositories and accession number(s) can be found at: GSA, CRA007596.

## Author contributions

HH: conceptualization, supervision, and project administration. X-GH and YX: methodology. X-GH and HZ: software. X-GH, YX, and NS: validation. NS: formal analysis. PB: investigation. X-GH and PB: data curation. X-GH: writing-original draft preparation, visualization, and funding acquisition. X-GH, EL, and ZT: writing-review and editing. All authors contributed to the article and approved the submitted version.

## Funding

This project was supported by Zhejiang Natural Science Foundation of China (LQ21C160002), National Science Foundation for Young Scientists of China (Grant No. 32001327), Zhejiang University Student’s Science and Technology Plan and the New Talent Program (2021R412003), Key Scientific and Technological Grant of Zhejiang for Breeding New Agricultural Varieties (2021C02070-1), and Key research and development project of Zhejiang Province (2021C02037).

## Conflict of interest

The authors declare that the research was conducted in the absence of any commercial or financial relationships that could be construed as a potential conflict of interest.

The handling editor declared a shared affiliation with the authors at the time of review.

## Publisher’s note

All claims expressed in this article are solely those of the authors and do not necessarily represent those of their affiliated organizations, or those of the publisher, the editors and the reviewers. Any product that may be evaluated in this article, or claim that may be made by its manufacturer, is not guaranteed or endorsed by the publisher.
