# Supplementary figures and images for: Comparative physiological analyses and the genetic basis reveal heat stress responses mechanism among different Betula luminifera populations
Source: Front Plant Sci. 2022 Sep 23;13:997818. doi: 10.3389/fpls.2022.997818 (PMC9538328; doi:10.3389/fpls.2022.997818)

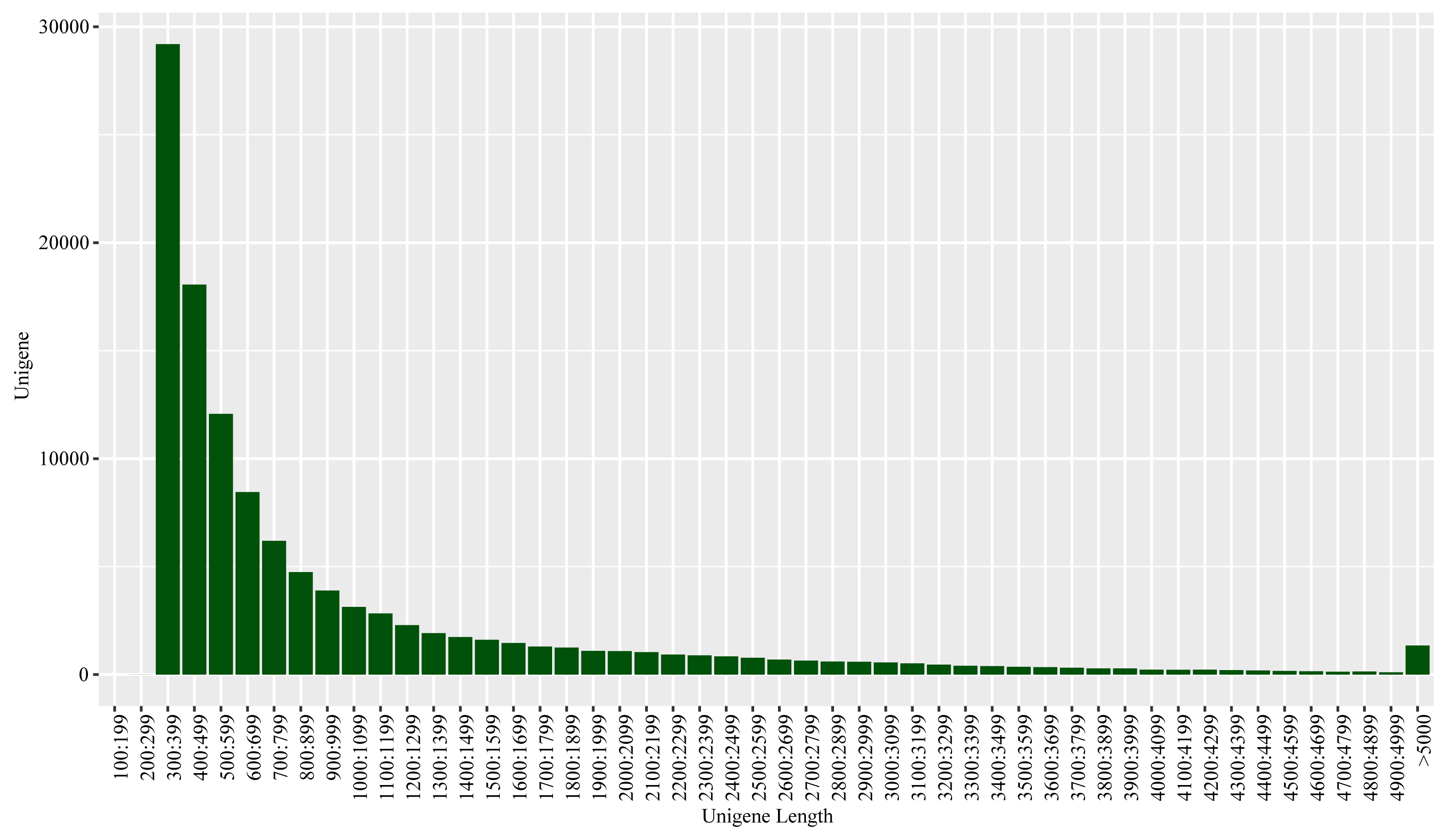

Supplement: Supplementary file 1 [file Image_1.TIF]

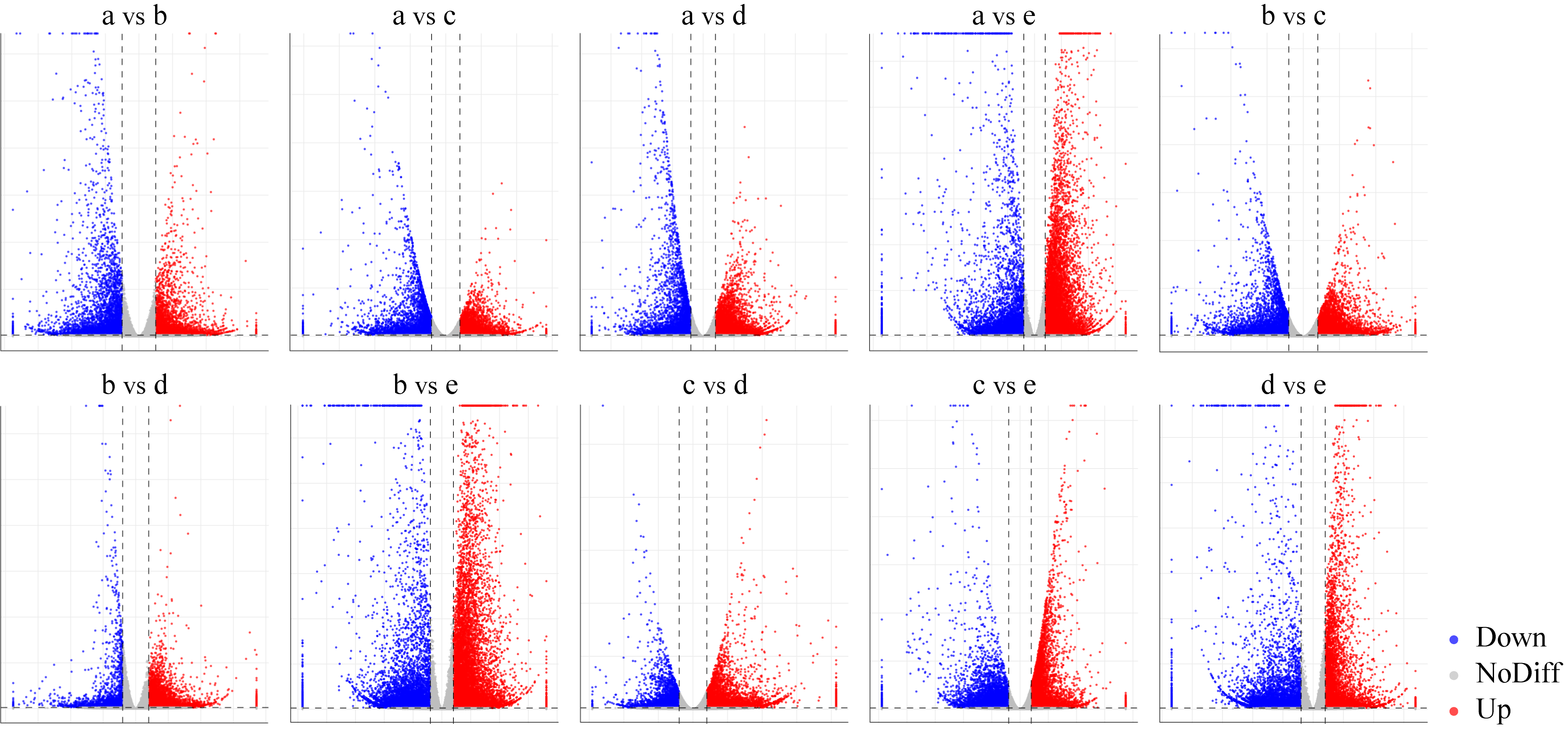

Supplement: Supplementary file 2 [file Image_2.TIF]

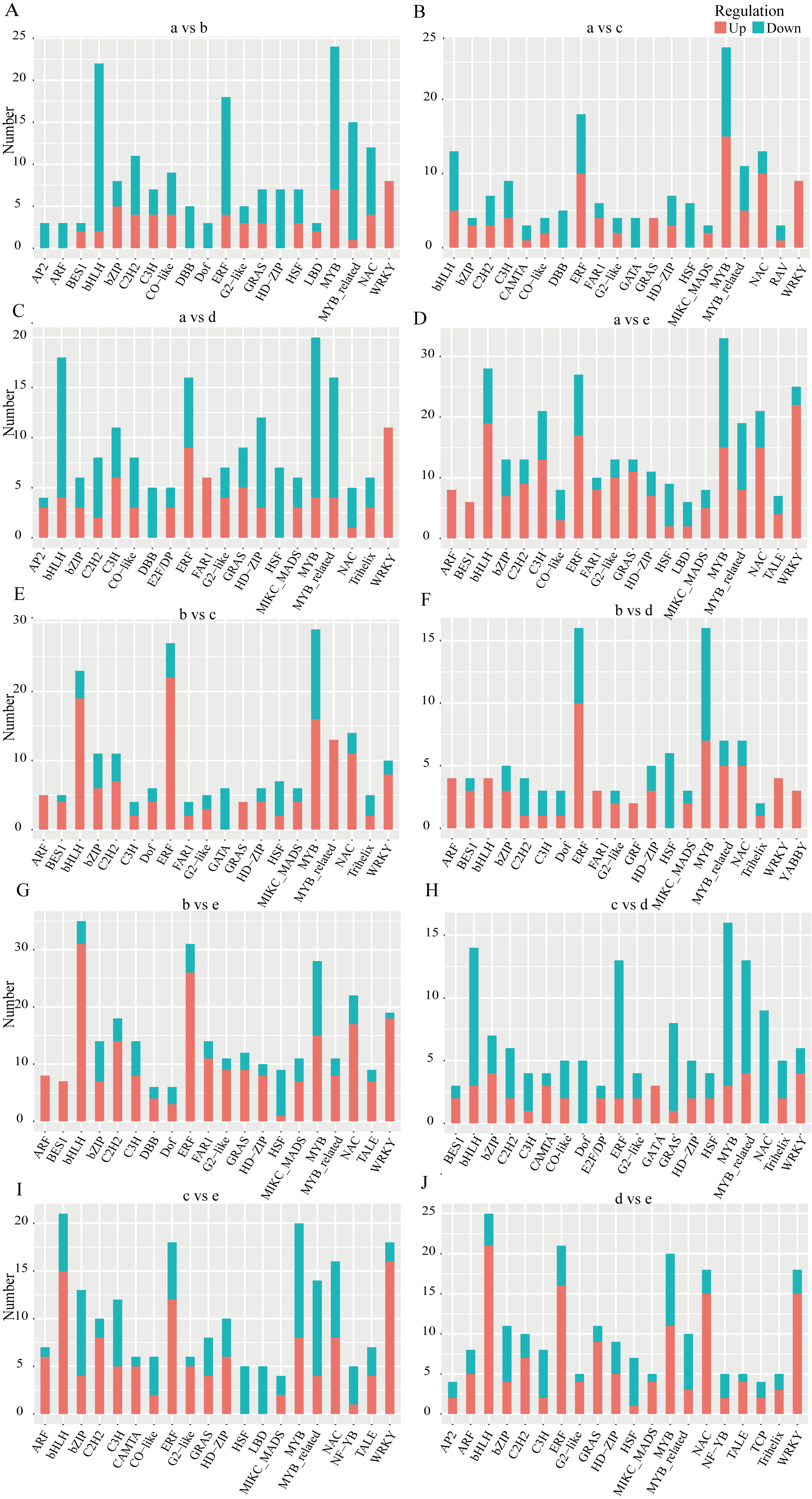

Supplement: Supplementary file 3 [file Image_3.TIF]
